# The Problem of Meaning: The Free Energy Principle and Artificial Agency

**DOI:** 10.3389/fnbot.2022.844773

**Published:** 2022-06-23

**Authors:** Julian Kiverstein, Michael D. Kirchhoff, Tom Froese

**Affiliations:** ^1^Academic Medical Center, Amsterdam, Netherlands; ^2^Amsterdam Brain and Cognition, University of Amsterdam, Amsterdam, Netherlands; ^3^Faculty of Arts, Social Sciences, and Humanities, School of Liberal Arts, University of Wollongong, Wollongong, NSW, Australia; ^4^Embodied Cognitive Science Unit, Okinawa Institute of Science and Technology Graduate University, Okinawa, Japan

**Keywords:** artificial agency, sensorimotor autonomy, the free energy principle, active inference, problem of meaning, frame problem, relevance problem

## Abstract

Biological agents can act in ways that express a sensitivity to context-dependent relevance. So far it has proven difficult to engineer this capacity for context-dependent sensitivity to relevance in artificial agents. We give this problem the label the “problem of meaning”. The problem of meaning could be circumvented if artificial intelligence researchers were to design agents based on the assumption of the continuity of life and mind. In this paper, we focus on the proposal made by enactive cognitive scientists to design artificial agents that possess sensorimotor autonomy—stable, self-sustaining patterns of sensorimotor interaction that can ground values, norms and goals necessary for encountering a meaningful environment. More specifically, we consider whether the Free Energy Principle (FEP) can provide formal tools for modeling sensorimotor autonomy. There is currently no consensus on how to understand the relationship between enactive cognitive science and the FEP. However, a number of recent papers have argued that the two frameworks are fundamentally incompatible. Some argue that biological systems exhibit historical path-dependent learning that is absent from systems that minimize free energy. Others have argued that a free energy minimizing system would fail to satisfy a key condition for sensorimotor agency referred to as “interactional asymmetry”. These critics question the claim we defend in this paper that the FEP can be used to formally model autonomy and adaptivity. We will argue it is too soon to conclude that the two frameworks are incompatible. There are undeniable conceptual differences between the two frameworks but in our view each has something important and necessary to offer. The FEP needs enactive cognitive science for the solution it provides to the problem of meaning. Enactive cognitive science needs the FEP to formally model the properties it argues to be constitutive of agency. Our conclusion will be that active inference models based on the FEP provides a way by which scientists can think about how to address the problems of engineering autonomy and adaptivity in artificial agents in formal terms. In the end engaging more closely with this formalism and its further developments will benefit those working within the enactive framework.

## Introduction

The problem of meaning has haunted artificial intelligence (AI) more or less from its inception, and it still hasn't been solved. It goes by a variety of names: the symbol grounding problem; the frame problem; and the relevance problem, and it stands behind John Searle's famous Chinese room thought experiment (Searle, 1980). In what follows we will take the problem to be how to engineer artificial agents that are the source of their own values, needs and goals. Such an agent will have its own perspective relative to which its engagements with the world are imbued with meaning.

We take as our starting point Froese and Ziemke's (2009) biological principles for the design of artificial agents. First they argued for a shift in the design process toward engineering the appropriate conditions for an agent to self-generate and sustain its own identity as an individual agent under precarious conditions—a property we refer to as “autonomy” (Thompson, 2007; Di Paolo and Thompson, 2014; Di Paolo et al., 2017). Autonomy is a property of the organization of living systems that is introduced to explain how such systems can be self-individuating. Biological systems possess autonomy when the processes that make up the system form an “operationally closed” set of mutually enabling relations. The organization of the system as a whole is constantly regenerated by the activities of its constituent processes. In the absence of any of the co-enabling relations among its constituent processes, the organization of the system would break down, and is therefore described as “precarious”.

The second design principle they proposed is that artificial agents should exhibit “adaptivity”: the process by which an autonomous system regulates its interaction with the environment so as to avoid situations that would lead to a loss of viability, were they to be encountered. Froese and Ziemke argued that an agent that exhibits this dual profile of autonomy and adaptivity would have its own point of view on the world. Relative to this point of view, actions can be evaluated as good or bad, adequate or inadequate, successful or unsuccessful for maintaining the organism's viability.

In practice, it has proven difficult to design artificial agents that satisfy the first condition of being physically self-individuating. An alternative strategy, first proposed by Di Paolo (2003), has therefore been to design agents that acquire regular, relatively stable, and self-sustaining patterns of sensorimotor engagement with their environment (Egbert and Barandiaran, 2014; Di Paolo et al., 2017; Ramírez-Vizcaya and Froese, 2020). Instead of building robots that instantiate metabolic processes that self-organize to form autonomous networks, the strategy has been to build robots whose *sensorimotor* processes self-organize to form autonomous networks. Such stable, self-sustaining patterns of sensorimotor interaction, are the basis for what we will call “sensorimotor autonomy”. The organization of sensorimotor behavior can ground the values, norms and goals necessary for an artificial agent to encounter a meaningful environment in much the same way as biological autonomy does in living systems.

It is this strategy for solving the problem of meaning in artificial agents that we take up in this paper. We will consider whether the Free Energy Principle (FEP) might provide formal tools for modeling the conditions required for an agent to acquire sensorimotor autonomy. The FEP states that organisms act to keep themselves in their expected phenotypic and ontogenetic states, and they achieve this goal by minimizing an information-theoretic quantity referred to as “free energy”. In this specific sense, the FEP implies that all living systems can be modeled as if they visit a bounded and limited set of states (but not necessarily the exact same states) if they are to continue to exist (Friston, 2019). Active inference describes the process of selecting actions that minimize free energy over time. Could active inference models based on the FEP be used to mathematically model sensorimotor autonomy?

We argue first that the FEP can be applied to many systems that do not satisfy the conditions for sensorimotor autonomy, such as swinging pendulums and Watt governors (Kirchhoff and Froese, 2017; Kirchhoff et al., 2018; Baltieri et al., 2020). One can model such systems as inferring the hidden states of their observations, and thereby treat them as if they were engaged in updating their posterior distributions in accordance with Bayesian inference. We go on to distinguish physical systems like synchronizing pendulums that can be modeled as engaging in “mere” active inference from systems that are modeled as engaging in what we will call “adaptive active inference”. Adaptive active inference refers to the process of actively selecting actions that minimize *expected* free energy associated with their future states (Kirchhoff et al., 2018). Mere active inference allows one to give a description of coupled systems (e.g., swinging pendulums) as inferring the hidden states of one another, thus updating their posterior beliefs. However, this is only a description. Moreover, in mere active inference, the relevant systems cannot actively change their relation to their environment. It is good to be able to update one's beliefs about the world; it is even better to be able to actively change one's relation to one's environment. It is this crucial latter aspect that is captured by shifting from mere active inference to adaptive active inference. As an example of a model of adaptive active inference, we describe a recent simulation of bacterial chemotaxis (Tschantz et al., 2020). Chemotaxis is often given as a flagship example of adaptivity. Tschantz et al. showed how their simulated agent could learn to engage in chemotaxis by means of processes of *expected* free-energy minimization. We go on to argue that adaptive active inference may well provide formal tools for modeling sensorimotor autonomy (drawing on previous work by Kirchhoff et al., 2018; Ramstead et al., 2021; van Es and Kirchhoff, 2021).^1^

Our aim in this paper is to argue that the FEP could potentially serve as a modeling technique for designing artificial agents in accordance with enactive principles. We seek to use the FEP to provide enactive cognitive science with formal tools for modeling sensorimotor autonomy. Such a research programme must however confront a number of significant challenges that have emerged in the recent literature. We take up two in what follows.

First, it has recently been argued that biological systems are not well described as state-determined systems that over time are attracted toward non-equilibrium steady-states (Froese and Taguchi, 2019; Aguilera et al., 2021; Di Paolo et al., 2022). These authors have argued that organisms (perhaps in contrast to FEP-based models of agency) have a natural history that is characterized by open-ended, unpredictable transitions to qualitatively new regimes of order. Di Paolo et al. (2022, p. 21) give as examples “embryogenesis, life-cycle patterns, epigenetic variability, metamorphosis, and symbiosis.” They argue furthermore that qualitative transformations can be observed in the structure of behavior in the learning of skills, and in the soft assembly of task-specific systems in tool use (Anderson et al., 2012; Di Paolo et al., 2017). These critics have argued that processes of historical change are essential to adaptivity but such history-dependent processes cannot be captured in the terms of the FEP. Once a system returns to a non-equilibrium steady-state its history is forgotten. If these critics are correct, there are therefore essential differences between systems that engage in adaptive active inference, and biological agents that exhibit sensorimotor autonomy.

Second, Aguilera et al. (2021) have argued that a free energy minimizing system would fail to satisfy a key condition for sensorimotor agency referred to as “interactional asymmetry”. They show how the mathematical assumptions the FEP rests upon only apply to systems whose interactions with the environment are symmetrical. If Aguilera et al. are correct, the mathematics of the FEP is not well suited for modeling sensorimotor autonomy. The FEP doesn't take us any further forward in understanding the formal properties of systems that are the source of their own values, needs and goals.

We finish up by offering reasons why the door should remain open to a synthesis of the FEP and enactive cognitive science we propose in our paper. First, we argue that the FEP is highly general, applying to both systems that implement mere active inference as well as to systems that are able to perform adaptive active inference. We suggest this generality is an advantage of the FEP allowing it to approximately represent a wide range of different systems including, if the arguments of our paper hold up, systems that fall in the region of those possessing sensorimotor autonomy. Second, we will argue that systems that implement adaptive active inference will tend to exhibit transient or metastable dynamics in which there is a recurring creation and destruction of large-scale coordination dynamics. Although metastable systems can be described as on average revisiting their attracting states they will avoid ever settling into any of these attracting states. Metastable systems exhibit the kind of historical, path-dependent learning required for acquiring a sensorimotor identity, and becoming an agent. Thus the key question for the FEP is whether adaptive active inference can be used to model systems with metastable dynamics. We will provide reasons for returning a positive answer to this question; though the work of building such formal models, so far as we know, remains to be done.

We conclude that the two frameworks need each other. Enactive cognitive science needs the FEP to formally model the properties it argues to be constitutive of agency. The FEP needs enactive cognitive science for the solution it provides to the problem of meaning. In the end engaging more closely with this formalism and its further developments will benefit those working within the enactive framework.

### The Enactive Approach to the Problem of Meaning in Artificial Intelligence

Biological agents are able to act in ways that express a sensitivity to context-dependent relevance. Organisms engage with an environment that is structured by their practical involvements, cares and concerns. Minimally, organisms have a concern for their own continued existence and their manners of living. Organisms must for instance engage in a continuous struggle to stave off death. Human agents are of course concerned with much more than meeting basic biological needs required for survival. Their activities are animated and driven by a variety of desires they strive to satisfy, many of which stem from distinctively human, sociocultural ways of living.

The problem of meaning arose in artificial intelligence in attempting to design artificial agents that are able to act adaptively and flexibly in dynamic complex and open-ended real-world situations. A popular approach in artificial intelligence research has been to build systems that learn an internal model of their environment and that make inferences and plans on the basis of this internal model (e.g., Lake et al., 2017). The sensitivity to what is relevant in a perceived situation has however proved resistant to specification in ways that could allow for this sensitivity to be captured in an internal model. To act adaptively and flexibly in dynamic complex environments such a system will need to determine from its internal model what is actually relevant under conditions of continuous change. Everything the system knows is of possible relevance. How then does the system determine what is of actual relevance without engaging in an exhaustive search of everything it knows (Dennett, 1984; Dreyfus, 1992; Fodor, 2000; Wheeler, 2005; Samuels, 2010; Vervaeke et al., 2012; Vervaeke and Ferraro, 2013; Danks, 2014; Shanahan, 2016)? The problem could perhaps be solved if artificial intelligence researchers could find a way to make an internal model that represents all possible contexts in terms of their determinate properties. However, the environment that living beings encounter in perception is not an environment made up of objects and properties that stand in determinate logical relations. As we began this section by noting, organisms perceive an environment that is structured by their needs, cares and concerns.

In what follows we will take the problem of meaning to be equivalent to what is sometimes called the “relevance problem.” Thus, we will use the terms “meaning” and “relevance” interchangeably in what follows. Meaning and relevance should be distinguished from information which we take to refer to statistical correlation between states of two systems (e.g., an organism and its environment). We take it to be uncontroversial that statistical correlation does not suffice to make it the case that the states of a system are meaningful for the system (Hutto and Myin, 2013). In living systems what makes a state the bearer of meaning is the history, dynamics and structure of the system (Varela, 1979; Oyama, 2000; Thompson, 2007). The history and structure of the living organism serve as the basis for needs, goals and values that move the organism to act in its environment. Meaning is determined by the organism's history, dynamics and structure. We identify meaning with relevance because we take meaning to be brought forth by the agent through a history of engagement with an environment that is relevant to the agent because it is structured by the agent's needs, concerns and values.

Froese and Ziemke (2009) argued that the problem of meaning could be circumvented if artificial intelligence researchers were to design agents based on the assumption of the continuity of life and mind. The core idea behind life-mind continuity is that intelligence depends upon its biological embodiment, where embodiment is to be understood in terms of the organizational properties of autonomy and adaptivity. The idea of continuity is therefore that the concepts and principles required for understanding and explaining features of mind such as subjectivity, agency and individual identity, are also the principles and concepts employed to explain the phenomenon of life (Kirchhoff and Froese, 2017; Di Paolo, 2018: p.74). Agents that are biologically embodied are the source of their own norms, values and goals. They escape the problem of working out from everything they know, what is relevant to their current and future contexts of activity. Relevance is not an extra ingredient that has to be added to what the agent already knows but is instead intrinsic to what is perceived. To see how this follows, we must further unpack the key concepts of biological autonomy and adaptivity briefly encountered in our introduction.

To possess biological autonomy a system must first of all be operationally closed. That is to say it must be organized so as to produce “a network of precarious processes in which each process enables at least one other process in the system and is, in turn, enabled by at least one other process in the system” (Di Paolo et al., 2017: p.113). The operationally closed network has a precarious existence insofar as the constituent parts that make up the network as a whole, are processes that stand in co-enabling relations. Each process would break down were it not causally enabled by the other processes in the network. The component processes are co-enabling insofar as they work together to produce the larger network as a whole. The self-production of the network as a whole is a task that needs to be continually accomplished if decay, disintegration and death are to be avoided. The system can therefore be said to be biologically autonomous in the sense that it is the operation of the processes that make up the system that enable its continued self-production and its self-distinction from its surroundings.

Systems that are biologically autonomous constitute or produce themselves as individuals – they are self-individuating.^2^ This process of self-individuation serves as the basis for agency – the organism is able to distinguish and actively regulate flows of energy and matter that contribute positively to its self-individuation, and avoid those that potentially interfere with its biological autonomy (Varela, 1991; Di Paolo, 2005; Thompson, 2007). The organism's coupling with its environment is inherently risky because of the precariousness of the processes that produce and sustain the organism's continued existence. To succeed in its goal of continuously realizing processes of self-production, the organism must be selectively open to energetic exchanges with the environment that contribute to the conditions of its self-production, and closed to exchanges that threaten its self-distinction (Di Paolo, 2018: p.84). Autonomy thus underwrites a basic biological form of normativity – the capacity to differentiate between, and thereby regulate, flows of matter and energy based on how well or badly they contribute to the organism's goal of maintaining its precarious identity. The classic example of biological normativity, and one we will return to later, is chemotactic behavior in which a bacterium will move toward metabolisable compounds and move away from metabolic inhibitors.

The biological normativity that is intrinsic to autonomy is not dependent on the observer's perspective on the organism's behavior. It is a capacity that is intrinsic to the organism's biological organization. The organism's capacity to regulate and modulate its relation to the environment is dependent on a sensitivity to dynamical trajectories, gradients, and tendencies Di Paolo has labeled “adaptivity”. Adaptivity is agentive in the sense that it is a capacity the system actively exercises in changing the parameters and conditions of the agent-environment relation in for instance seeking out food when energy is anticipated to be needed. This active modulation introduces an asymmetry into the organism's coupling with its environment, referred to in the literature as “interactional asymmetry” (Barandiaran et al., 2009; Di Paolo et al., 2017, §5.2.2). The organism modulates its relation to the environment based on its sense of whether environmental events are good or bad for its continued existence.

Di Paolo (2005) defines adaptivity as:

“A system's capacity, in some circumstances, to regulate its states and its relation to the environment with the result that, if the states are sufficiently close to the boundary of viability,

1. Tendencies are distinguished and acted upon depending on whether the states will approach or recede from the boundary and, as a consequence,
2. Tendencies of the first kind are moved closer to or transformed into tendencies of the second and so future states are prevented from reaching the boundary with an outward velocity.” (Di Paolo, 2005: p. 438)

The reason this is important is because it implies that the organism need not passively respond to environmental events in a state-determined manner based only on its previous state. The organism's operating conditions can undergo change over time based instead upon its history of interactions with its environment. The organism can in this way have a plastic identity that is given shape by its history of acting, and being acted upon by its environment. Meaning can thus be understood as actively generated or brought forth by the organism based on the history of sensorimotor interaction with its environment that has become sedimented in its biological organization. So conceived, meaning does not need to be somehow added to what the organism knows because the environment the organism relates to is always already imbued with meaning based on the organism's past history of interaction.^3^

So far our treatment of the concepts of biological autonomy and adaptivity has focused on processes that the organism depends upon for its continued viability. However, the norms relative to which the organism regulates its interactions with the environment do not only concern its continued existence in the here and now. The significance of sensory perturbations for the organism go beyond their immediate bearing upon the organism's operationally closed organization.^4^ The processes that constitute and produce the organism as an *agent*, include its regular and relatively stable patterns of sensorimotor behavior. These patterns of sensorimotor behavior have been argued to also exhibit the key properties of autonomy—they depend upon operationally closed sensorimotor networks made up of co-enabling bodily and environmental processes (Di Paolo, 2005; Barandiaran, 2008; Egbert and Barandiaran, 2014; Di Paolo et al., 2017; Ramírez-Vizcaya and Froese, 2020). Think for example of habits like smoking cigarettes or drinking coffee when you wake up in the morning. These are sensorimotor patterns of behavior that are self-sustaining, but that do not positively contribute to maintaining the organism's biological viability, and may even be harmful to the organism. A pattern of behavior becomes a self-sustaining habit when the processes that enable it—neural, muscular and environmental—depend for their stability and organization on the regular performance or enactment of the pattern of behavior (Di Paolo et al., 2017: p.144; also see Egbert and Barandiaran, 2014). Thus these processes come to form operationally closed sensorimotor networks in much the same way as metabolic processes do. At the same time, the organization of the sensorimotor network is precarious because it is at risk of extinction if the pattern of behavior is not regularly enacted.

It has recently been proposed that sensorimotor autonomy could serve as a design principle for artificial agents that would allow researchers to avoid the difficult problem of engineering systems that metabolically self-produce. Di Paolo (2003) suggested for instance that robots could be built with mechanisms “for acquiring a way of life, that is, with habits” (p.31). Designing agents that can acquire self-sustaining habits will have the consequence that such agents will engage with the world based on norms, goals and values that relate to the sustaining of their habits. They will differentially evaluate the situations they encounter in terms of their relevance for the realization of processes upon which the sustaining of their habits depend. Such an agent doesn't relate to an action-neutral world that stands in need of representation. It will not need to work out from all possible responses, which responses are actually relevant to its current situation. Instead agent and environment will form a single system that is continuously reconfigured in ways that allow for the sustaining of the sensorimotor autonomy of the agent. This is, in a nutshell, the enactive proposal for how to solve the problem of meaning in artificial intelligence.

Still a question remains of how to model sensorimotor autonomy. The question we take up in the rest of our paper is: could the free energy principle (FEP) provide a formal description of the conditions for the design of an artificial agent that possesses sensorimotor autonomy?

### The Free Energy Principle: A Brief Introduction

The FEP purports to describe the organizational properties a system must instantiate if it is to preserve its organization over time in its interaction with a dynamic environment. The FEP has been argued to apply to “any biological system…from single-celled organisms to social networks” Friston and Stephan (2007). It claims that all complex adaptive systems that are able to resist a tendency to disorder must minimize an information-theoretic quantity known as “free energy”. Friston (2010) for instance formulates the FEP as follows:

“The free-energy principle… says that any self organizing system that is at equilibrium with its environment must minimize its free energy. The principle is essentially a mathematical formulation of how adaptive systems (that is, biological agents, like animals or brains) resist a natural tendency to disorder.” (Friston, 2010: p.127).

The FEP is sometimes described as a tool the scientist employs purely for modeling purposes. Raja et al. (2021) for instance formulate the FEP as claiming: “Any ergodic random dynamical system with an attractor and a Markov blanket behaves *as if* it were minimizing the variational free energy of its particular states” (p.3, our emphasis). The “as if” qualifier here is used to indicate that the behavior of complex adaptive systems is modeled on the assumption that adaptive systems minimize variational free energy. It doesn't matter for modeling purposes if this assumption is true. A number of papers argue on this basis that strictly speaking the FEP has nothing to say about the organizational properties of the complex adaptive systems it purports to model (Ramstead et al., 2020b; van Es and Hipólito, 2020; van Es, 2021). These authors argue the FEP should be understood in purely instrumental terms as a scientific tool for predicting the observable behavior of adaptive systems. Our paper is premised on the assumption that such an instrumentalist reading of the FEP is incorrect [for further discussion see Andrews, 2021; Kirchhoff et al., 2022; Kiverstein and Kirchhoff, 2022]. Our aim in this paper is to consider whether the FEP can be used to formally model sensorimotor autonomy. We take sensorimotor autonomy to be a real organizational property that tells us what it is for a system to be an agent. Our aim is to consider if models based on the FEP can be taken to truthfully represent a real organizational property of agents.

The FEP, as we will understand it, employs the mathematical formalism of non-equilibrium steady-state (NESS) systems to model the properties a complex adaptive system must instantiate if it is to preserve its organization over time (Friston, 2012, 2013, 2019). Any biological system will be able to maintain order within a boundary (modeled as a “Markov blanket,” more on which below), separating the internal states of this system from the external states of its environment. The FEP claims that to maintain order within this boundary, the system must (on average, and over time) revisit a set of sensory states when it is perturbed by the environment. We will refer to the set of sensory states that the system is modeled as repeatedly revisiting over time as the “attracting set” for a given biological system. We can think of the attracting set as a model of the system's extended phenotype since it will include variables for morphological states as well as behavioral patterns that relate to the niche the agent constructs (Friston, 2011; Kirchhoff and Froese, 2017; Bruineberg et al., 2018; Kirchhoff and Kiverstein, 2019). A system's attracting set will include physiological states such as blood oxygen concentration and pressure levels and body temperature that must be maintained within a certain range of values if the organism is to survive. Other sensory states belonging to a system's attracting set relate to its niche - fish frequent aquatic environments, while humans tend to live on land and only occasionally find themselves underwater. The states belonging to the system's attracting set will therefore be the subset of all possible states the system can occupy that are highly probable given the system's phenotype and the niche it inhabits. States that fall outside the attracting set are potentially threatening to the maintenance of order within the system because they lead to an increase in disorder or entropy within the system. States that lead to an increase in disorder will be surprising or improbable for a NESS system that tends toward an ordered set of states over time in its exchanges with the environment. The states belonging to the system's attracting set are states the system expects to occupy over time. When the states of the system fall outside of its attracting set this is therefore surprising because the probability of finding the system in such states is low. (“Surprise” is to be understood as the improbability of a particular sensory state, and is not to be confused with agent-level surprise, which occurs in response to an unexpected conscious sensation).

The system has no tractable way of calculating whether a given sensory state is surprising or not. This is because the probability of a sensory state is calculated relative to a state of the possible influences of external states of the environment on the internal states of the system. The state space is however potentially infinite, thus computing the probability of each sensory state by searching through this state space will prove intractable. This is where free energy can help, since free energy is a quantity that can act as an upper bound on surprise. Free energy more technically is a function of the function of sensory states that is parameterized by the internal states of a system. Since free energy is a function of the sensory and internal states of the system, it is in principle computable (Friston and Ao, 2012). Moreover, it is a quantity over which the organism has (indirect) control since it maps onto the organism's sensory states that it can control through action, and internal states that admit of a certain degree of plastic reorganization through learning. Minimizing free energy will guarantee that sensory states remain in a high-probability area in the system's state space. So long as the NESS system can keep the free energy associated with its sensory states to a minimum, it will succeed in remaining in states that belong to its attracting set.

The FEP states that all quantities that can change in a NESS system will change to minimize free energy (Friston and Stephan, 2007). Free energy quantifies the mismatch between the sensory states the system *expects* to sample through its actions, and those it actually samples. The notion of “expectation” should be understood in relation to a model that is entailed by the internal dynamics that form in the system's coupling with its niche. The function of this model is to anticipate sensory perturbations originating in the environment external to the system, allowing the system to proactively adapt its actions to those perturbations.

The FEP models complex adaptive systems as random dynamical systems that are attracted toward a non-equilibrium steady-state (a NESS). The FEP assumes adaptive systems will tend to exhibit certain dynamical flows of states over time determined by, amongst other things, their phenotypic states, body morphology, and their ecological niche. Generative models are used to describe the statistics of these flows (Ramstead et al., 2020a). For a system to tend to flow toward a NESS by minimizing free energy is for the system to minimize the discrepancy between the variational density (also sometimes called the “recognitional density”) the organism instantiates in its internal dynamics, and the true posterior or the external dynamics in the environment. Free energy can thus be thought of as quantifying mathematically the mismatch between the organism's internal dynamics and the external dynamics of its environment (Bruineberg et al., 2018).

Friston (2013) has proposed that a living system does not have a model of its environment but it is a model of its environment, which highlights that the notion of “model” the FEP is premised upon is implicit in the living system's internal dynamics. In this sense, there is no distinct system inside of the central nervous system of the agent that uses a model to engage in inference. For Friston, inference just is a description of the flow of the internal dynamics of the living system. Friston takes the generative model to be organized around the organism's belief in its own continued existence. All of the actions the organism undertakes aim at sampling sensory states that maximize the evidence for this belief in its continued existence, a belief Allen and Tsakiris (2018) have referred to as the “first prior”. To minimize free energy is at one and the same time to maximize evidence for this belief in the living system's continued existence. Hohwy (2016) refers to this property of living systems whereby they act to sample evidence that confirms the belief in their own continued existence as “self-evidencing”.

Free energy can be minimized in two intimately related ways referred to as “perceptual” and “active inference”. In perceptual inference free energy is reduced by changing the dynamics internal to the system (Friston, 2010; Hohwy, 2013). The internal dynamics of the system embody a model of the agent's econiche by means of which it can steer its actions (Friston, 2011). Perceptual inference involves plastically restructuring the internal dynamics in such a way that the agent is better able to accommodate external sensory perturbations arising from the changes in its niche in the future (Friston et al., 2016). Free energy is kept to a minimum in part by generating and modifying an internal dynamics that closely approximates the external environmental dynamics. We said that perceptual and active inference are intimately related (Hohwy, 2013). This intimate relation follows from what we have just referred to as self-evidencing (Hohwy, 2016): the internal dynamics that are adjusted in perceptual inference are organized around sampling sensory evidence that confirms the agent's belief in its own continued existence (Fotopoulou and Tsakiris, 2017; Allen and Tsakiris, 2018; Seth and Tsakiris, 2018).

In active inference the agent acts to sample sensory states belonging to its attracting set (Friston et al., 2017a,b). The sensory states that are expected given the first-prior are those that relate to the agent's needs, goals and intentions (Allen and Tsakiris, 2018). The agent's continued existence will for example depend on its meeting its biological needs for warmth, nourishment, and attachment (Fotopoulou and Tsakiris, 2017). If the agent is to sample sensory states that maximize the evidence for the first prior, this will require the agent to act in ways that satisfy such basic needs. A simple example is eating when hungry. Hunger indicates a potential breach of essential variables relating to blood glucose levels (i.e., a deviation from the system's attracting set). The action of eating helps to correct this potential breach before it arises. With this brief summary in place we turn next to the question of whether the FEP provides a formal description of the conditions required for an artificial agent to possess sensorimotor autonomy.

### The Free Energy Principle: A Minimal Condition for Sensorimotor Autonomy?

Recall our proposal is to use sensorimotor autonomy as a biologically-based design principle for building artificial agents (Barandiaran, 2008; Egbert and Barandiaran, 2014; Di Paolo et al., 2017; Ramírez-Vizcaya and Froese, 2020). The idea is that habits are self-sustaining patterns of activity that constitute a systemic identity for the agent relative to a sensorimotor domain. Relevance arises out of the needs, goals and interests the agent has in sustaining its habits. Situations and activities “become meaningful not only in virtue of their contribution to biological survival, but also in virtue of their contribution to the stability and coherence of a sensorimotor repertoire” (Di Paolo et al., 2017, p.39). An agent that has sensorimotor autonomy will have its own point of view relative to which evaluations of action possibilities can be made in terms of their relevance for the agent. Does the FEP provide a set of mathematical tools that can be used to model sensorimotor autonomy? Is free energy minimization sufficient for sensorimotor autonomy? The payoff for a positive answer to this question will be formal tools that allow us to connect meaning and relevance to a system's intrinsic dynamics.^5^

The FEP is broad in terms of the systems to which it applies. Swinging pendulums, Watt governors and pebbles have all been argued to count as systems that can be described as minimizing free energy in their dynamic coupling with the environment (Kirchhoff and Froese, 2017; Kirchhoff et al., 2018; Baltieri et al., 2020; van Es and Kirchhoff, 2021). Two coupled pendulums A and B can, for example, be described as modeling each other's motion. Given the internal states of pendulum A, and the effects of its velocity and motion on the beam from which it is hanging, pendulum A can be said to infer the motion of pendulum B. This is possible because the motion of pendulum A, through its effects on the beam, enslaves the motion of pendulum B, and vice versa. When the two pendulums come to swing in synchrony the coupling of the two pendulums can therefore be described in terms of free energy minimization (Bruineberg et al., 2018; Kirchhoff et al., 2018). In line with our earlier work, we describe this process of free energy minimization that can be observed in non-living, and non-cognitive systems as “mere active inference” (Kirchhoff et al., 2018). Each pendulum infers through its own motion and the effects of its motion on the beam, the motion of the other pendulum.

Mere active inference is qualitatively different from the process of free energy minimization that occurs in living and cognitive systems (Kirchhoff, 2018). Living systems are able to sample among different options, and select the option that has the least *expected* free energy.^6^ While the pendulums enslave each other's motion, living systems are able to free themselves from their proximal conditions by selecting temporally extended sequences of actions that minimize expected free energy associated with future states. We have used the term “adaptive active inference” to describe what living systems are able to do that is missing in systems that engage only in mere active inference (Kirchhoff et al., 2018). In adaptive active inference sequences of actions are selected that minimize the cumulative sum of free energy over time, a quantity referred to as “expected free energy” (Friston et al., 2017a,b).

Adaptive active inference is distinguished from mere active inference in aiming at the selection of actions whose sensory effects minimize expected free energy. (Expected free energy is the free energy expected upon executing a temporally-extended sequence of actions.) Expected free energy is a function of two quantities referred to as instrumental and epistemic value.^7^ To minimize expected free energy an agent must select action policies (sequences of actions) that maximize both instrumental and epistemic value. Instrumental value is maximized when the sensory observations an agent expects to sample match its preferred outcomes (its needs, goals and desires). Thus, acting to maximize instrumental value can be thought of as equivalent to goal-directed behavior. Epistemic value quantifies information gain or the reduction of uncertainty about the hidden states of the environment. An agent maximizes epistemic value by maximizing the information that is gained through exploratory actions of the environment. An active inference agent that acts to minimize expected free energy will continuously be balancing instrumental actions that aim at bringing about preferred outcomes with epistemic actions that aim at uncertainty reduction. Crucially, while this kind of epistemic (or information seeking) foraging should *on average* result in the minimization of uncertainty, there will nevertheless be short-term peaks of uncertainty given an organism's exploration of its surroundings. The aim is thus to strike the right balance between the reduction of entropy and temporarily increasing entropy. The pay-off for finding this right balance (what is sometimes called the “exploitation-exploration trade-off”) is that the agent will avoid getting trapped in any local minima. They will be able to make continuous progress and improvements in learning in ways that are conducive to long-term free energy minimization. (For further discussion see Kiverstein et al., 2019).

There are other points of importance to note about adaptive active inference. First, the generative model is biased toward sampling sensory observations that match the agent's preferences, goals and desires (Bruineberg et al., 2018; Tschantz et al., 2020). Second, and relatedly, epistemic actions will work in the service of tinkering with a model that is biased toward the control of certain sensory outcomes. As Tschantz et al. have noted, an active inference agent will tend to forage for information in parts of the environment expected to maximize instrumental value (Tschantz et al., 2020: p.7). That is to say, the improvement in the model that epistemic actions make possible are ultimately improvements in the service of the agent's goals.

To minimize expected free energy the agent has to select from among action policies, the policy that is expected to lead to preferred outcomes and goals (Friston et al., 2017a,b; Pezzulo et al., 2018). This might be thought to lead the active inference agent to encounter the relevance problem once again.^8^ The agent will always be faced with an open-ended range of possible action policies but can only search a narrow area within this space. How then does the agent constrain the search space to only action policies of relevance (i.e., those expected to minimize free energy? Most active inference models up until now have avoided this question by pre-specifying the search space. Within this predefined search space action policies are then selected on the basis of the agent's belief in the precision of the policy - the confidence the agent places in the sensory consequences of its actions. The work of scoring action policies is taken over by the precision estimate associated with each action policy. Precision estimates are based on expected uncertainty (or salience) and unexpected uncertainty (or volatility, Parr and Friston, 2017). The higher the precision for each action policy, the more confident the agent can be that the sensory outcomes of its action will match its preferred outcomes. A risky action policy is one whose sensory consequences the organism anticipates will diverge from its preferred outcomes leading to an increase in expected free energy. Precision estimates can be thought of as having effects comparable to attention. They bias action selection toward actions whose sensory consequences are expected to minimize free energy. The “gain” is turned-up on opportunities to bring about those sensory consequences. Precision is decreased and the gain turned-down on actions whose sensory consequences are associated with increases in free energy.

Is there reason to believe that adaptive active inference will scale-up from a predefined search space of action policies, without the agent once again encountering the relevance problem?^9^ Recall how we are proposing that artificial agents that develop sensorimotor autonomy will circumvent the problem of meaning. Meaning will arise out of the agent's history of activity in an environment structured by its needs, interests and concerns. Meaning is not an extra ingredient the agent needs to add to information to determine how to solve what would otherwise be an ill-defined problem. “With ill-defined problems, the goal-state is often murky, the initial state is unclear,” and the operations that will take you from your initial state to your goal state are unspecified (Vervaeke and Ferraro, 2013, p.4). Before one can solve an ill-defined problem one must determine what information is relevant for defining the problem. Our hypothesis is that agents that possess sensorimotor autonomy however will typically not encounter ill-defined problems.^10^ They will relate to an environment that is already meaningful because of their past history of engagement. The habits they have developed provide them with know-how or skills that form the basis for norms that guide the agent's actions. Situations and activities are good or bad, adequate or inadequate, successful or unsuccessful to the extent that they contribute to the sustaining of the agent's sensorimotor identity.

We will consider next if models of adaptive active inference could be used to formally describe the organizational property of sensorimotor autonomy. To address this question we need to briefly introduce the Markov blanket formalism. The terminology of Markov blankets is borrowed from the literature on causal Bayesian networks (Pearl, 1988; Bruineberg et al., 2022). The Markov blanket for a node in a Bayes network comprises the node's parents, children and parents of its children. The behavior of the blanketed node can be predicted from the states of the blanket without knowing anything about the nodes external to the blanket that are the causes of changes internal to the network. We suggest the Markov blanket formalism can be used to model sensorimotor autonomy. Here we make the case only informally and schematically. It is a task for future research to turn our philosophical argument into concrete formal models.

Our core idea is that the autonomy of the sensorimotor network can be modeled as the nesting relations among Markov blankets in systems that perform adaptive active inference. Each component process in the system can be thought of as having its own Markov blanket. Two components A and B stand in an enabling relation when the active states of the Markov blanket of A cause the sensory states that belong to the attracting set of B (i.e., the sensory states that B must occupy if it is to remain viable). B will begin to break down when the sensory states that form its Markov blanket are improbable, departing from what is expected given its attracting set. Thus B's continued viability is enabled by the active states of A. Conversely, component B enables component A if the sensory states belonging to A's attracting set are made highly probable by B's active states. So long as the Markov blankets of each of the component processes couple in such a way that each of the components remains in high probability sensory states, (a condition that will be satisfied in systems that engage in adaptive active inference) the result will be the self-production and self-distinction of the system as a whole (Ramstead et al., 2021; van Es and Kirchhoff, 2021). A system that engages in adaptive active inference will succeed in maintaining operational closure under precarious conditions.

Nave (2022) criticizes the use of Markov blankets to model metabolic self-production. She argues that organisms are intrinsically unstable structures that define their boundaries while undergoing near constant material turnover. To deploy the Markov blanket formalism we would first need to identify the organization of the system of interest, which is a challenge in living systems undergoing continuous material change. She concludes that the Markov blanket formalism can only be successfully deployed if we already know the organization of the system we are interested in modeling. Along similar lines, Raja et al. (2021) have argued that while the cell membrane is the product of the activity of cells, the Markov blanket is not the product of the activity of a cognitive system's internal states. They conclude: “There is nothing in the use of Markov blankets that accounts for the fundamental features of the boundary of self-organized, self-maintained systems” (p.28-9; cf. Suzuki et al., 2022).

We suggest in response that the self-production of living systems is understood as an example of autonomy (i.e., the production and maintenance of an operationally closed network under precarious conditions). Such a characterization of the organization of living systems fits perfectly with Nave's description of organisms as “intrinsically unstable structures - stabilized only via their own ceaseless activity” (Nave, 2022, preprint, p.4), and with Raja et al.' concept of constitutive self-organization. We have just shown informally how the Markov blanket formalism could be applied to systems that are modeled as engaging in adaptive active inference. To repeat the main idea: the sensory states that define the Markov blanket for each component of an operationally closed system will be coupled to the active states of one or more of its enabling components. So long as the system engages in adaptive active inference this coupling relation will ensure that the sensory states for each component belong to the component's attracting set. The result will be the self-production of the system as a whole as a unity distinct from its environment.

As a proof of concept example of how adaptive active inference can be used to model sensorimotor autonomy (but not biological autonomy), consider the recent active inference simulation of chemotaxis of Tschantz et al. (2020). “Chemotaxis” refers to the running and tumbling movements bacteria exhibit when they encounter a chemical gradient that is a potential source of food (i.e., a sucrose gradient). This can be thought of as a form of pragmatic action in which the bacterium acts to maximize instrumental value. When bacteria sense a negative gradient (i.e., an acid that is toxic to the bacterium), the rhythm of the running and tumbling motions alters in such a way as to steer the bacterium away from danger, and in search of locations were positive gradients are to be found. This behavior can be thought of as an epistemic action the bacterium performs to maximize epistemic value.

Tschantz et al. simulated an active inference agent that selected between actions by seeking to maximize both instrumental and epistemic value. They showed that in their simulation agents employing such a strategy were able to perform at least some chemotaxis (i.e., running toward positive gradients, and tumbling away from negative gradients). The strategy of minimizing expected free energy seems to have allowed the active inference agent to find the right balance between performing epistemic exploratory actions of tumbling and instrumental actions of moving forward. The agent engaged in tumbling behaviors when it estimated there was less instrumental value in running. In doing so it learned about the effects of tumbling, and continued to do so until the value of tumbling becomes less than the value of running when the agent switches its behavior.

Crucially, the value the simulated agent assigned to actions was modeled by the change in free energy over time. The policy of tumbling for instance decreases in value when the agent is no longer making information gains that resolve model uncertainty, a situation that can be understood in terms of free energy remaining constant or increasing. The policy (i.e. sequences of actions expected to minimize FE) of running takes on a value that outweighs that of tumbling when the agent expects sensory observations that match those it prefers (i.e., a positive gradient). The increased instrumental value of running can therefore be equated to an expected reduction in free energy. This is important because valence has been analyzed and modeled in the FEP literature in terms of change in free energy over time (Joffily and Coricelli, 2013; Van de Cruys, 2017; Kiverstein et al., 2019; Hesp et al., 2021). “Valence” refers to the positive or negative charge of an affective state.

The rate of change in free energy can be taken as a measure of how well or badly the organism is faring in its interactions with the world. When free energy is on the increase, or is not resolved through action, this means that the agent is in a potentially threatening situation, while when free energy is decreasing this is feedback for the agent that it is faring well and should, if possible, continue on the same path. We suggest then that Tschantz et al.' active inference agent exhibits adaptivity in its chemotactic behavior. The active inference agent uses changes in free energy to negotiate the trade-off between performing epistemic and pragmatic actions, as we have just explained. The changes in free energy over time are used by the agent as feedback that signals how well it is doing in its goal of achieving chemotaxis, and the simulated agent modulates its coupling with its environment on the basis of this feedback. In the next section we take up two objections that challenge the hypothesis we have been proposing that the process of adaptive active inference can be used to model sensorimotor autonomy.^11^

### Ergodicity, Historicity and Interactional Asymmetry

The first objection we will consider targets the ergodicity assumption that early iterations of the free energy principle relied upon (e.g., Friston, 2013). Briefly, “ergodicity” refers to “the time average of any measurable function of the system converges (almost surely) over a sufficient amount of time. This means that one can interpret the average amount of time a state is occupied as the probability of the system being in that state when observed at random.” (Friston, 2013, p. 2) If ergodicity holds, the proportion of time a system spends in any region of its phase space is equivalent to the probability of the system occupying this region of its phase space. For example, if the probability of a coin landing heads is 50/50 then over the course of the time spent flipping a coin, the coin will spend 50% of this time landing heads, and 50% of this time landing tails. We can think of the average time a system spends in any region of its phase space – the space of all possible states of the system – as being proportional to the attractiveness of that region. Recall the idea of an attracting set, that living systems as random dynamic systems, will have a set of sensory states toward which they will continually evolve over time whenever they are perturbed. This idea has been taken by critics to be based on the assumption that living systems literally are ergodic.^12^

It has recently been argued that the enactive concept of adaptivity is fundamentally at odds with the ergodicity assumption (Di Paolo et al., 2022; also see Colombo and Wright, 2018; Kauffman, 2019 for a critique of ergodicity as applied to living systems). Adaptivity, they have argued, involves changes in the phase space of the dynamical system the organism forms with the environment to avert the potential loss of viability that would ensue, were the agent to remain in a steady-state regime. The possibility of such critical transitions in an organism's phase space requires an understanding of the change in internal dynamics the agent undergoes as path-dependent, that is, as dependent on the agent's history of interaction with the environment. We see examples of such phase transitions in development, in for example, “embryogenesis, life cycle patterns, epigenetic variability, metamorphosis and symbiosis” (Di Paolo et al., 2022: p.21). In behavior, critical transitions occur in perceptual learning, skill acquisition, tool use and habit formation. Over shorter time scales, changes in patterns of effective connectivity in the brain that allow for many-to-many mapping between neural structure and function, or what Anderson (2014) calls “neural reuse”, depend upon such critical transitions. In short, phase transitions are ubiquitous in living and cognitive systems. Di Paolo et al. characterize adaptivity in terms of phase transitions. An adaptive act is, they contend, a phase transition in which an agent undergoes a change in structure switching from an existing dynamical trajectory that would lead to a loss of viability eventually if left unchecked. The history of an organism can be described as the “cumulative change” in the configuration of the phase space that describes the behavior of the organism over the course of its lifetime.

Di Paolo et al. argues that this characteristic of path-dependence, whereby the agent's internal dynamics are dependent on its past history of phase transitions, is fundamentally incompatible with the idea of an attracting set of non-equilibrium steady-states to which the organism repeatedly returns when perturbed. A system that conserves its organization in this way will, they argue, quickly forget its history. The long-term average of the states the system visits over time will be equivalent to the averaging of the states in an ensemble of the system at a time. Di Paolo et al. take this to describe a key difference between physical systems that tend to conserve invariant structure and biological systems that rely upon a continuous reconfiguration of their structure following critical transitions. If adaptivity happens in such moments of critical transition, it would seem to follow that adaptivity cannot be understood in terms of adaptive active inference.

First, let us agree with Di Paolo et al. that adaptivity does indeed occur in moments of critical transition in the dynamics of an organism-environment system (cf. Varela, 1995). Indeed historical path-dependence has been central to how we have analyzed meaning in this paper. We suggest the appearance of incompatibility of adaptivity, so conceived, with the FEP may stem from the generality of the FEP. Recall how the FEP is equally applicable both to physical systems that engage in mere active inference, and to biological systems which engage in adaptive active inference. The past history of dynamical interaction is indeed irrelevant to describing how the swinging pendulums enslave each other over time. However, the path independence of behavior is less obviously true of systems that exhibit adaptive active inference.

Recall that such systems are able to strike the right balance between the reduction of expected free energy through instrumental actions, and temporarily increasing free energy through exploration of the environment. To strike a balance between exploitation and exploration an adaptive active inference agent will need to instantiate a metastable dynamics. Metastability is the consequence of two competing tendencies (Kelso, 1995): the tendency of the parts of the system to separate and express their own intrinsic dynamics, which leads to an increase in free energy, and the tendency of the parts to integrate and coordinate to create new dynamics, in the way that Di Paolo et al. argue is required for adaptivity. Metastable systems are able to transit between regions of their phase space spontaneously without external perturbation. The structure of a metastable system is therefore transient. Systems with metastable dynamics avoid getting trapped in fixed-point attractors that lead to a single outcome. The internal dynamics are instead itinerant or wandering in a way that allows for exploratory behaviors that temporarily increase free energy (Zarghami and Friston, 2020). However, such temporary increases in free energy allow for just the kind of dynamical reconfiguration that Di Paolo et al. take to be essential for adaptivity.^13^

Indeed we suggest that systems that can find the right balance between reducing and temporarily inducing increases in entropy would need to be capable of dynamically reconfiguring their internal dynamics in ways that fit with the context in which they are acting. This is not to deny that the internal dynamics of an adaptive active inference agent can never become rigid and inflexible over time. However such rigidity is perhaps a signature feature of psychopathologies (cf. Carhart-Harris et al., 2014). Think for instance of obsessive compulsive disorder in which the agent finds themselves trapped in maladaptive cycles of behavior. What is characteristic of such pathological behaviors is a weakening of metastable dynamics that in healthy individuals allows for finding the right balance between reducing and increasing entropy.

To summarize our response to Di Paolo et al., we have argued that an agent that exhibits adaptive active inference will exhibit the historical path-dependence of behavior they take to be required for adaptivity. Such an agent will need to exhibit path-dependent behavior if it is to succeed in maximizing both the instrumental and epistemic value of its action policies. Indeed, any system that learns a model of its environment will exhibit plastic changes in its internal dynamics. The appearance of an incompatibility between the enactive approach to life and cognition and the FEP stems from the generality of the FEP. Certainly some of the systems to which the FEP applies will not be capable of adaptivity (e.g., those that are modeled as performing mere adaptive inference) but it doesn't follow that no systems the FEP is used to model could exhibit adaptivity.

We turn next to a second recent paper that also challenges our proposal to use adaptive active inference to formally model sensorimotor autonomy. It has been argued that to apply the mathematics of the FEP to concrete physical systems requires specific assumptions that do not typically apply to the sensorimotor interactions of living systems (Aguilera et al., 2021). Aguilera et al. argue for the opposite conclusion from the one we have been defending, that the FEP is highly particular in the systems to which it applies. Indeed they claim the FEP is so particular in its requirements as to fail to pick out the class of systems that would qualify as having sensorimotor autonomy. Aguilera et al. make their argument by considering the assumptions that would be required to apply the FEP to a class of simple systems whose dynamics are described by stochastic linear differential equations. They select such systems on the grounds that if the assumptions of the FEP do not apply to such simple systems, it is unlikely that they hold for more complex non-linear systems.

Aguilera et al. begin by considering the type of sensorimotor interface that, according to the FEP, mediates the interaction of the internal dynamics of the agent and the external dynamics of the environment.^14^ They show that the sensorimotor interface must have two statistical properties. First, they must be described by the Markov blanket formalism, whereby internal and external states are conditionally independent given the sensory and active states of a Markov blanket. Second, the sensorimotor interface must be such that solenoidal couplings between internal and external states are decoupled by blanket states. Aguilera et al. define “solenoidal couplings” as arising from “dissipative tendencies in the system” that drive a system “away from equilibrium” (Aguilera et al., 2021: p.2). They show that any system that possesses a sensorimotor interface satisfying these two statistical properties will exhibit an internal dynamics that can be described in terms of descent on a free energy gradient. Aguilera et al. show that to connect the average flow or internal dynamics of a system with a gradient minimizing free energy requires the assumption that a Markov blanket precludes solenoidal couplings between internal and external dynamics.

The no solenoidal couplings (NSC) assumption raises difficulties for our claim that adaptive active inference is sufficient for adaptivity. Aguilera et al. show that a system that conforms with the NSC assumption will possess a sensorimotor interface that precludes adaptivity. This is because systems that satisfy the NSC assumption will possess a sensorimotor interface that permits only fully symmetrical interaction loops to form between agents and environments. If systems that conform with the FEP must exhibit fully symmetrical sensorimotor interactions with the environment, such systems will lack adaptivity. For adaptivity, as we have seen above, requires interactional asymmetry between agent and environment. Adaptivity requires that the agent be able to modulate its interaction with the environment in such a way as to influence the constraints on the agent's behavior, where some of these constraints are due to the agent, and others to its environment.^15^

By way of a reply, we begin by briefly considering more carefully the claim that the sensorimotor interface implied by the FEP can be modeled as a Markov blanket that induces a separation described statistically in terms of conditional independence of internal and external states. Now it is crucial to note that the Markov blanket is not fixed once and for all but the sensory and active states out of which it is built continuously undergo change, based on the agent's coupling with its environment. The accumulation of fluctuations will gradually render the states of the Markov blanket independent of the initial conditions that gave rise to them. Given sufficient time, the FEP implies that a system that minimizes expected free energy should instantiate a probability density that converges on a NESS. However in the intervening period of time as fluctuations accumulate, internal and external dynamics enter into a transient state of conditional dependence mediated by the Markov blanket. Thus, the Markov blanket condition, that is the conditional independence of internal and external dynamics, is temporarily violated. This violation of the Markov blanket condition has been argued to allow for memory (Parr et al., 2021) but we suggest it should also allow for a modulation of the agent-environment relation in line with interaction asymmetry.

This takes us back to our earlier discussion of the historical path dependence of behavior. Recall that it was the capacity of agents that conform with the FEP to modulate the parameters and constraints on their coupling with the environment that was in contention in this earlier discussion. We argued that neural processes that alter their dynamics in fluid and adaptive ways, in response to the requirements of particular contexts of activity, are part and parcel of adaptive active inference. Such neural processes are an essential part of selecting action policies that maximize instrumental and epistemic value in a dynamical environment. The model of chemotaxis of Tschantz et al. already exhibits a bistable dynamical profile. It is able to endogenously switch between running and tumbling based on changes in free energy. We take this simulation as a demonstration that an agent can be formally described in accordance with the FEP and exhibit a minimal form of sensorimotor agency.

Aguilera et al. may respond that our argument fails since systems that satisfy the NSC assumption *must* engage in symmetrical sensorimotor interactions with the environment. We suggest however that the systems that the FEP models are dynamical systems that can temporarily violate the assumptions the models rest upon, while at the same time on average and over time conforming to those assumptions. Aguilera and colleagues ask what assumptions are needed to apply the equations of FEP to a specific class of systems whose dynamics are described by stochastic linear differential equations. Such an argument seems to assume however that in order for the FEP to be used to represent the dynamics of physical systems, its mathematical equations must literally be instantiated by those physical systems. This is an example of what we have elsewhere called the “literalist fallacy”—the fallacy of taking the properties of FEP models to literally map onto real-world target systems (Kirchhoff et al., 2022). We suggest instead that active inference models based on the FEP are better conceived of as idealisations and approximations that introduce deliberate distortions. The Markov blanket assumption is an example of such a distortion, which is why the systems that the FEP describes can violate this assumption, while at the same time FEP based models can accurately represent the longer-term dynamics of such systems.

Similar arguments can be made in response to the argument of Di Paolo et al. that systems with an attracting set or NESS are memoryless, and are therefore incapable of historical path-dependent behavior. Di Paolo et al. critique trades on the assumption that in order for the FEP to truthfully represent a system, the properties it models must literally be instantiated by a system. We have been arguing however that the systems the FEP purports to model are dynamical systems that can fruitfully be represented as tending to evolve toward states belonging to their attracting set. The FEP can serve as the basis for models that provide truthful but approximate and idealized representations of such systems, including systems that instantiate sensorimotor autonomy, if the arguments of our paper are valid. We conclude with some additional issues for further research.

## Conclusion

Artificial intelligence from its earlier days has struggled with the problem of meaning. The information that computers process does not mean anything *for* the system that is doing the processing. This information only means something for the users of these systems. We have argued that the imperative to minimize expected free energy could serve as an intrinsic norm for an artificial agent. Thus adaptive active inference could provide a formal description of the conditions an artificial agent would need to satisfy to possess sensorimotor autonomy and thus to perceive a meaningful environment (see Kolchinsky and Wolpert, 2018 for a related proposal). We finished up by considering two objections to our thesis that the imperative to minimize expected free energy may serve as an intrinsic norm for an agent. These objections generate a number of important questions for further research, which we will end by highlighting.

First, we have argued that models of adaptive active inference can be used to formally describe systems that possess sensorimotor autonomy. However it could be objected that such an agent could indeed be considered a *model* of sensorimotor autonomy but without itself possessing this property. Just as a model of intelligence may lack intelligence, similarly a model of sensorimotor autonomy may not itself instantiate this property. To genuinely instantiate such a property, it might be argued that an agent would need to have a material body composed of processes that self-organize to form operationally closed networks, and that distinguish the agent as a unified individual from its environment. The artificial agent of Tschantz et al., which we have taken as our main example in this paper, has no material body but exists only *in silico*. When it is simulating chemotaxis, it does not engage in exchanges of matter and energy with its environment that are part of its process of self production and self differentiation. Thus no matter how good a model of autonomy and adaptivity it may be, it might be argued it does not yet possess these organizational properties.

Second, and relatedly, Froese and Taguchi (2019) have argued that modeling autonomy and adaptivity will fail to solve the problem of meaning. They concede that artificial agents may be simulated that act *as if* they have their own intrinsic norms. They argue however that an important disanalogy will remain with organic life. An organism actively brings about its own existence through engaging in metabolic activity. Its continued existence or being is, in an important sense, a consequence of its own doings. It is this relationship between being and doing that makes for goals and concerns that are intrinsic to the organism. Froese and Taguchi (2019) argue that any simulation of artificial agency cannot be said to genuinely have a existence that is the consequence of its own doing. They argue that there is no room for meaning, normativity or value to make a difference to the behavior of such agents insofar as they act in a simulated environment that is fully deterministic. The behavior of a simulated agent is due to dynamical constraints on its internal and interactional dynamics, not to the agent's bringing forth a domain of meaningful action. Froese and Taguchi argue on this basis that if meaning is to make a real difference to the behavior of an agent, some indeterminacy must be built into the agent's engagement with its environment.

Finally, more work is needed on the challenges that arise from applying the mathematics of the FEP to concrete sensorimotor agents. Are systems whose dynamics are describable in terms of non-equilibrium steady-states also capable of path-dependent behaviors, as we have argued? If the application of the FEP to concrete systems depends upon the NRC assumption, as Aguilera et al. show, does it follow that all systems describable in terms of the FEP must engage in symmetrical interactions with their environment? Can the FEP be used to model systems with metastable dynamics? We argued that these are related challenges but more work is certainly required on the implications of answering them for the FEP. While there is a good deal more work to be done, we have argued that the synthesis of enactive ideas with the FEP may set biologically inspired AI research on a promising path for addressing the problem of meaning.

## Data Availability Statement

The original contributions presented in the study are included in the article/supplementary material, further inquiries can be directed to the corresponding author.

## Author Contributions

JK wrote the manuscript. MK and TF provided critical feedback and editing suggestions. All authors contributed to the article and approved the submitted version.

## Funding

JK was supported by grants awarded to Erik Rietveld from the Dutch Organisation for Scientific Research and the European Research Council H2020 Starting Grant (number 679190). MK was supported by the Australian Research Council Discovery Project (DP170102987).

## Conflict of Interest

The authors declare that the research was conducted in the absence of any commercial or financial relationships that could be construed as a potential conflict of interest.

## Publisher's Note

All claims expressed in this article are solely those of the authors and do not necessarily represent those of their affiliated organizations, or those of the publisher, the editors and the reviewers. Any product that may be evaluated in this article, or claim that may be made by its manufacturer, is not guaranteed or endorsed by the publisher.

## Footnotes

^1^Note that it remains an open question what the precise relation is between the simulation-based chemical agent and actual chemical agents. There are good reasons to think that in models of active inference agents, the similarity is sufficiently tight to infer properties about actual chemical agents on the basis of simulation-based models of chemical agents (see Kirchhoff et al., 2022 for further defense of this claim).

^2^Maturana and Varela's (1980) concept of autopoiesis is a paradigm example of biological autonomy. An autopoietic system is composed of co-enabling processes that form an operationally closed network, and that dynamically produce the system's own material boundary. It should be noted that systems can be autonomous while lacking a material boundary. This is the case for insect colonies for instance that form autonomous social networks (Thompson, 2007: p.44).

^3^Von Uexküll (1957) introduced the idea of a sensorimotor world (an *Umwelt*) to characterize this meaningful environment. The *Umwelt* is the world as it is perceived by an organism given its sensorimotor repertoire.

^4^Di Paolo et al. (2017) refer to Margaret Donaldson's distinction between four modes of human thinking about the world, which she distinguished based on the degree of decentering from the agent's point of view on the world in the here and now. Humans can for instance plan for future events, and they can detach themselves entirely from their own concerns in thinking about moral and political values. Di Paolo et al. appeal to what they call “virtual actions” to account for the development of these decentered modes of thinking out of sensorimotor agency (see also Kiverstein and Rietveld, 2018). It is beyond the scope of our paper to discuss their account of virtual actions in further detail.

^5^Kolchinsky and Wolpert (2018) have recently explored a strikingly similar proposal. They begin their paper by noting the difference between what they call “semantic information” and Shannon information as a measure of statistical correlation between two systems. They seek to provide formal tools that describe how semantic information could be intrinsic to the dynamics of a system in a given environment. Central to their proposal is a viability function which they define as “the negative Shannon entropy of the distribution over the states of the system X” (p.2). They use Shannon entropy as an upper bound on the probability that the system occupies states belonging to its viability set in a similar fashion to how Friston uses variational free energy in relation to a system's NESS. An important difference is that Shannon entropy is not computationally tractable for the agent, whereas variational free energy represents a quantity an agent can control through its actions. We will postpone exploring further the similarities and differences in our proposals. What we wish to emphasize for now is that both our proposals aim to formalize meaning (Kolchinsky and Wolpert use the term “semantic information”) in terms of a quantitative measure of viability - in our case variational free energy.

^6^The distinction between mere and adaptive active inference is formally grounded. For discussion see Millidge et al. (2021), though they do not use our terminology of “mere” and “adaptive” active inference.

^7^For mathematical details of how epistemic and instrumental value are computed we refer the reader to Tschantz et al. (2020, pp.25–26), and Friston et al. (2017a). For discussion of the relation between variational and expected free energy see Millidge et al. (2021).

^8^Our thanks to an anonymous reviewer for raising this objection.

^9^For recent reviews of the application of active inference models in robotics see Lanollis et al. (2021) and Da Costa et al. (2022). We discovered these papers only after completing the writing of this manuscript and plan to discuss them fully in follow-up work.

^10^We do not claim that skilled agents never encounter ill-defined problems. We suggest exploratory or “epistemic” actions that aim at uncertainty reduction will provide an important part of the answer to how agents solve such problems when they do arise (see e.g. Friston et al., 2017b). It is an important question for further research whether agents engaging in epistemic actions to solve an ill-defined problem would once again encounter the relevance problem. How is it that agents performing epistemic actions to solve an ill-defined problem constrain the space of possible solutions they sample? This question is partially addressed by active inference models of curiosity and insight (see e.g. Friston et al., 2017a). Thanks to one of our reviewers for raising this problem.

^11^Our argument that agents with sensorimotor autonomy will circumvent the problem of meaning shares much in common with the account of relevance realization developed by John Vervaeke et al. in a number of publications (e.g., Vervaeke et al., 2012; Vervaeke and Ferraro, 2013). Vervaeke et al. understand relevance realization in terms of the self-organizing optimisation of trade-offs between opponent yet complementary learning strategies. An example is how relevance could be realized in relation to the goal of threat avoidance through optimizing the trade-off between fight and flight. Vervaeke et al. also frequently use the example of the trade-off between exploration and exploitation. A discussion of similarities and differences between our approaches is unfortunately beyond the scope of this article. However, see Hovhannisyan and Vervaeke (2021) for a recent account of how the concept of relevance realization could contribute to developing an enactive approach to humanistic psychology.

^12^The ergodicity assumption is employed as an approximation to model systemic behavior. It requires that a system returns to approximately the same states over time. The notion of approximate similarity however should not be mistaken for numerical identity.

^13^One of our reviewers objected that random dynamical systems that tend toward a NESS cannot possess metastable dynamics. Friston has however provided many models of active inference that in his words “provide a key connection to dynamical approaches to the brain that emphasize…metastability” (Friston, 2010, p.134), a connection that the reviewer takes to be excluded by the derivation of the FEP. We suspect the reviewer is confusing the mathematics that are used to derive the FEP, which may well contain equations that fail to capture metastability, with the use of the FEP to model systems with metastable dynamics. We will return to this point below in responding to the challenges raised by Aguilera et al. recent work.

^14^Aguilera et al. discuss a second assumption required for applying the FEP to concrete systems that “implies decoupling the actions of an agent from its history of previous states” (Aguilera et al., 2021, p.3). Their critique of this assumption is related to that of Di Paolo et al., discussed earlier in this section, but it also raises additional issues we cannot tackle in this paper but hope to return to in future work.

^15^Di Paolo et al. note that the individual agent need not always be the source of the modulation of its coupling. Other agents can also induce asymmetric changes in dynamical constraints resulting in a modulation of the individual's coupling with the environment (Di Paolo et al., 2017: p.120). We set aside this important complication here.
